# Highlighting nonlinear patterns in population genetics datasets

**DOI:** 10.1038/srep08140

**Published:** 2015-01-30

**Authors:** Gregorio Alanis-Lobato, Carlo Vittorio Cannistraci, Anders Eriksson, Andrea Manica, Timothy Ravasi

**Affiliations:** 1 Integrative Systems Biology Laboratory, Biological and Environmental Sciences and Engineering Division, Computer, Electrical and Mathematical Sciences and Engineering Division, Computational Bioscience Research Center, King Abdullah University of Science and Technology (KAUST), Ibn Al Haytham Bldg. 2, Level 4, Thuwal 23955-6900, Kingdom of Saudi Arabia; 2 Division of Medical Genetics, Department of Medicine, University of California, San Diego, 9500 Gilman Drive, La Jolla, CA 92093 USA; 3 Biomedical Cybernetics Group, Biotechnology Center (BIOTEC), Technische Universität Dresden, Tatzberg 47/49, 01307 Dresden, Germany; 4 Department of Zoology, University of Cambridge, Cambridge CB2 3EJ, England

## Abstract

Detecting structure in population genetics and case-control studies is important, as it exposes phenomena such as ecoclines, admixture and stratification. Principal Component Analysis (PCA) is a linear dimension-reduction technique commonly used for this purpose, but it struggles to reveal complex, nonlinear data patterns. In this paper we introduce non-centred Minimum Curvilinear Embedding (ncMCE), a nonlinear method to overcome this problem. Our analyses show that ncMCE can separate individuals into ethnic groups in cases in which PCA fails to reveal any clear structure. This increased discrimination power arises from ncMCE's ability to better capture the phylogenetic signal in the samples, whereas PCA better reflects their geographic relation. We also demonstrate how ncMCE can discover interesting patterns, even when the data has been poorly pre-processed. The juxtaposition of PCA and ncMCE visualisations provides a new standard of analysis with utility for discovering and validating significant linear/nonlinear complementary patterns in genetic data.

The last decade has seen a proliferation of Genome-wide Association Studies (GWASs) leading to novel and important biological discoveries, some of which have tremendous clinical relevance^1^. Such scientific advances have only been possible thanks to interdisciplinary endeavours aimed at making sense of huge amounts of genetic data. As genetic information continues to accumulate, the research community is in need of tools that can quickly and informatively inspect thousands of individuals and their associated genetic variants.

Principal Component Analysis (PCA), an unsupervised machine learning technique for *linear* dimension reduction commonly used in a variety of disciplines^2^ and introduced to population genetics by Cavalli-Sforza and his team^3^, has been a standard approach to identifying collections of genotyped individuals as populations, and quantifying the level of genetic similarity amongst them. Using PCA, it is possible to determine whether the data has some structure^4^, based on a linear transformation that uncovers, in a low-dimensional space (commonly with visualisation in two dimensions), the presence of patterns with higher orthogonal variance in the high-dimensional space.

In PCA, the data is projected onto a new coordinate system such that the greatest genetic variance between individuals lies on the first coordinate (Principal Component 1 or PC1), the second greatest variance lies on the second coordinate (Principal Component 2 or PC2), and so on^2^. It is important to note that since each PC is orthogonal to the others, in theory, the variances explained by the PCs are mutually uncorrelated. In addition to identifying distinct groups of individuals (e.g. populations or ethnic groups), PCA can be used to detect migration patterns^5^, i.e. whether individuals are the product of interbreeding between previously separated populations (admixture)^4^ and whether individuals in case-control studies stand out from others due to ancestry differences (stratification)^5^.

PCA is one of the most commonly employed algorithms because it is efficient (it extracts linear patterns within a low computational time), user-friendly (it is a parameter-free transformation, i.e., it is an algorithm that does not require the tuning of numerous parameters^4^,^5^) and has a relatively straightforward interpretation^6^,^7^. In practice, the PCA approach has been shown to be very powerful and reliable, although it suffers from two major drawbacks: i) the curse of dimensionality, i.e., the problem of finding information in datasets characterised by an overwhelming number of features over samples, which is a typical problem in population genetics; ii) difficulties associated with revealing nonlinear patterns hidden in a high-dimensional space.

As a consequence of these problems, PCA is occasionally unable to detect differences between groups of individuals, even with prior knowledge that such differences exist. There is also the case in which although we do not have such prior knowledge, dissimilarities characterised by some unknown nonlinear feature relationship may be present in the high-dimensional space, but because PCA is unable to detect them, they cannot be identified.

The impact of the above-mentioned inconveniences might be reduced by using non-centred Minimum Curvilinear Embedding (ncMCE), proposed here, as a method for visually inspecting population genetics datasets in a manner complementary to that of PCA.

Minimum Curvilinearity (MC), the principle behind ncMCE, suggests that curvilinear distances between samples (here the population individuals) may be estimated as pairwise distances over their Minimum Spanning Tree (MST), constructed according to a selected norm (Euclidean, correlation, etc.) in a high-dimensional feature space (here the genotype frequency space). The collection of all nonlinear pairwise distances forms a distance matrix called the MC-distance matrix or the MC-kernel, which can be used as an input in algorithms for dimensionality reduction, clustering, classification^8^,^9^ and more generally in machine learning. In the case of MCE, the MC-kernel is centred (this operation is neglected in the non-centred version of the approach, namely ncMCE) and its singular value decomposition is used to favour a sample projection onto a two-dimensional space for visualisation and analysis^8^,^9^ (see Fig. 1 for a thorough description of the algorithm and the second section of the Supplementary Information (SI) for more details). This description categorises MCE and ncMCE in the ‘machine learning zoo’ (expression borrowed from the computational complexity theory^10^) as a form of nonlinear and parameter-free kernel PCA. The approach was originally introduced in its centred version, which provided remarkable results in: i) visualisation and discrimination of pain patients in peripheral neuropathy, and the germ-layer characterisation of human organ tissues^8^; ii) discrimination of microbiota in molecular ecology^11^; iii) stage identification of embryonic stem cell differentiation based on genome-wide expression data^12^. In this third example, MCE ranked first in a study of the performances of 12 different approaches tested (evaluated on 10 diverse datasets). More recently, the non-centred version of the algorithm has been used to visualise clusters of ultra-conserved regions of DNA across eukaryotic species^13^ and as a network embedding technique for predicting links in protein interaction networks^9^, outperforming several other link prediction techniques.

The success of ncMCE when applied to various types of problems (it can be more time-efficient and often more discriminative than its centred version^9^), as well as its parameter-free nature, prompted us to apply it to population genetics data in order to explore whether this approach can provide insights that are complementary to those provided by PCA, thereby offering a hierarchical and nonlinear representation of the relationships between and within different populations.

## Results

**Figure 2:**
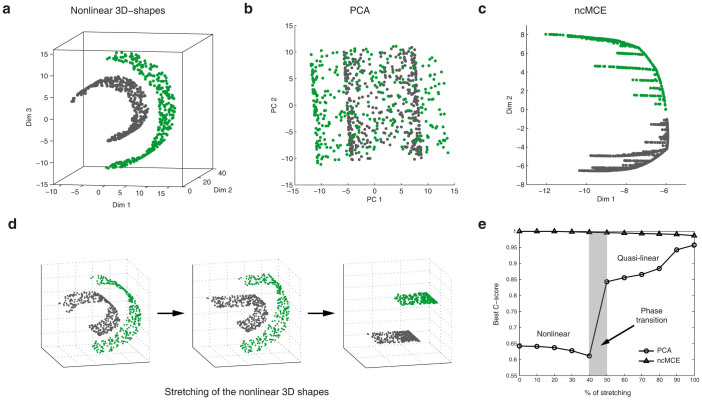
Linear and nonlinear projections of an artificial dataset. The correct embedding of the nonlinear clustered points of the artificial dataset presented in (a), requires the application of a nonlinear dimensionality reduction approach, like ncMCE (c), because the nonlinear structure of the data is not properly mapped to the low dimensional space using linear techniques, such as PCA (b). If the 3D shapes are gradually stretched until they form two planes (d), the nonlinear structure of the data is progressively linearised as indicated by an improvement of PCA's clustering quality in (e). Interestingly, while the behaviour of ncMCE is quite stable, giving always a well-defined separation, PCA presents a phase transition in the discrimination measure between the 40% and 50% of the stretching simulation-factor. This is a clear example of the instability of PCA in recovering patterns when it is not known, *a priori*, whether these patterns are nonlinear (see PCA curve when stretching factor is between 0 and 40%) or quasi-linear (see PCA curve when stretching factor is between 50% and 100%).

As a proof of concept, we first applied ncMCE to an artificial dataset to explore whether it could provide information complementary to that emphasised by PCA, for visually inspecting certain patterns hidden in datasets. Fig. 2a shows two clouds of points organised into two distinguishable nonlinear clusters in a three-dimensional feature space. Given the nonlinear relationship between these data points in three dimensions, their representation in two dimensions by a PCA projection failed to reveal the presence of the two clusters (Fig. 2b). The ncMCE projection, however, achieved perfect separation of the two clusters over the second dimension (Fig. 2c). Therefore, ncMCE highlighted very interesting nonlinear information that was hidden in the original three-dimensional feature space. This nonlinear information was not present in the output of the PCA transformation due to its linear nature. In this didactic example, we used a simple three-dimensional feature space to simplify the representation; however, the example is valid for any high-dimensional feature space: given the strong cluster nonlinearities of the data in the example, PCA would not be able to reveal the two clusters using any combination of principal components for projection^14^,^15^ (e.g., substituting the visualisation in PC1, PC2 with PCx, PCy, where x and y are any possible combination of reduced dimensions). If the artificial nonlinear shapes are linearised by gradually stretching them until they become planes in a three-dimensional space (Fig. 2d), an improvement of PCA's clustering quality, measured by computing the concordance score (C-score, see Methods for details) for dimension 1 and 2 and choosing the best, is clearly observed (Fig. 2e). PCA's performance presents a clear phase transition, which is commented in the caption of Fig. 2.

**Figure 3:**
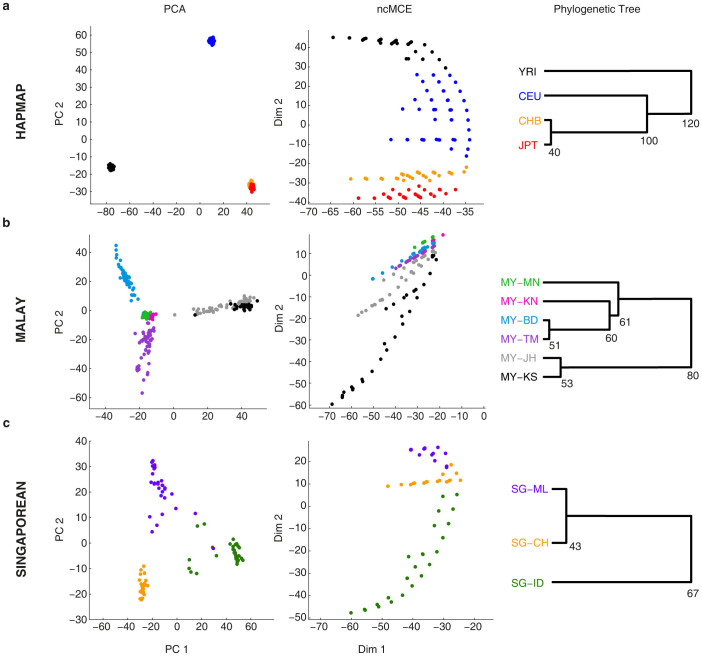
PCA and ncMCE complementarity. (a) PCA (left) provides a clear separation between the Yoruba (YRI), European (CEU) and Asian (CHB and JPT) samples but it is unable to detect the differences between the Chinese and Japanese individuals that form the Asian group. ncMCE (centre) clearly detected this difference over Dim2 and also provided an ordering over this dimension that was related to the organisation of these populations in a phylogenetic tree (right). (b) and (c): PCA (left) scattered the Malay and Singaporean individuals in a geographic manner. ncMCE (centre), just as in (a), clearly detected the genetic differences between individuals by separating ethnic groups over Dim2 and highlighting their phylogenetic relationships (right) over this same dimension. MY-MN and MY-KN are Malay Malay, MY-BD are Malay Bidayuh, MY-TM are Proto-Malay and MY-JH and MY-KS are Malay Negritos. SG-MY are Singaporean of Malay descent, SG-CH are Singaporean of Chinese descent and SG-ID are Singaporean of Indian descent.

As a first real world application of ncMCE, we analysed the HapMap panel comprising of four populations, one from Africa (Yoruba, YRI), one from Europe (CEU), and two from Asia (Chinese, CHB, and Japanese, JPT). For comparability with later examples, we use 54,794 SNPs covered by the Affymetrix GeneChip used for the Pan-Asian SNP Consortium Database (PanSNPdb)^16^,^17^. Fig. 3a shows an analysis, commonly employed in articles and tutorials, which illustrates the strengths and weaknesses of PCA applied to population genetics. PCA could distinguish the three continents, but was unable to simultaneously separate Japanese and Chinese individuals (JPT and CHB respectively). The ncMCE approach, on the other hand, identified a clear separation between all four populations over the second dimension of embedding (Dim2), placing the JPT and CHB samples close together but in separate clusters (see Fig. 3a, middle panel). The phylogenetic tree composed of these four populations (shown in Fig. 3a, right panel) predicted that the high degree of similarity between Asian individuals would make it difficult to separate them and also highlighted the ability of ncMCE to detect phylogenetic information in this data by ordering the populations immediately adjacent to their phylogenetically closer ethnicities (see the Methods section for details about the phylogenetic tree construction).

Next, we look at a regional example by comparing six Malaysian ethnic groups included in the PanSNPdb^17^. Fig. 3b shows a representative example of the geographic interpretation of PCA's axes of variation. PC1 mostly capture the latitudinal distribution of populations, with the Malay Negritos (MY-JH and MY-KS), which inhabit the North of the country, being assigned positive values and the other ethnic groups, found in the south, being assigned negative values. PC2 partially disentangles the southern groups, with MY-BD and MY-TM pulling apart and leaving MY-MN and MY-KN to form a tight, undifferentiated cluster. These ethnic groups are known to be genetically very similar^18^ (Fig. 3b, left panel), so it is not surprising that PCA failed to separate them (Fig. 3b, left panel, PC1). ncMCE, on the other hand, detected more separation (Fig. 3b, middle panel, Dim2), and this separation was hierarchically organised (Fig. 3b, right panel). This is a clear example in which the information provided by PCA and ncMCE was complementary.

We next look at three ethnic groups from Singapore (Fig. 3c). In this example, it is again possible to see how ncMCE complements PCA by extracting additional information. If PC1's most positive values are considered “west”, we can see how PCA scattered the Singaporean individuals according to their geographic origins (India, China and Malaysia, respectively, Fig. 3c, left panel). ncMCE, on the other hand, revealed clear genetic differences between the Singaporean samples by scattering them across three well-defined clusters over Dim2 (Fig. 3c, middle panel). Moreover, ncMCE's projection in this particular case coincided with the phylogenetic organisation of this population (Fig. 3c, right panel).

Taken together, these findings suggested a clear phylogenetic imprinting over the embedded dimensions provided by ncMCE, whereas PCA offered a more geographically oriented mapping. We suspect that the geographical information must be behind the data linearity, while the sparse and *tree-like* phylogenetic information must have an intrinsic nonlinear organisation.

**Figure 4:**
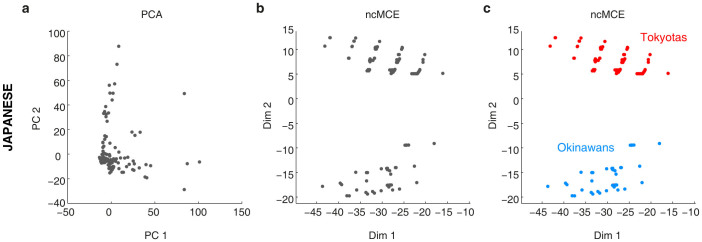
ncMCE finds additional patterns in population genetics data. Although PCA cannot reveal the presence of subgroups within the Japanese population (a), ncMCE clearly revealed defined sub-clusters (b). For the case of the Japanese individuals, we know that this separation is correct because Japanese from Tokyo (JPT & JP-ML) are different from those from Okinawa (JP-RK). This result is clearly revealed by ncMCE (c). The use of a single colour for all individuals in the PCA plot (a) would make it impossible to recognise the presence of the two sub-clusters.

The missing data problem is quite common in quantitative research studies, including population genetics^19^,^20^. The way in which missing SNP values are dealt with is so important, and its impact on techniques such as PCA can be so serious^4^, that various methods have been proposed to address this issue^19^,^21^. In the examples shown up to this point, missing SNP values in the genotype matrix have been imputed with the mode (most frequent value) for each specific SNP (see the Methods for more details), but we next show an example in which missing values remain in the dataset and ncMCE is still able to reveal population structure, whereas PCA is afflicted by this poorly pre-processed dataset. Fig. 4a and Fig. S3 in the SI show that PCA was unable to detect any clear separation between the Japanese ethnic groups of the PanSNPdb. Fig. 4b shows that ncMCE in fact revealed additional substructure within this population by separating Japanese individuals into two clear subgroups over Dim2. This was further confirmed by other two nonlinear dimensionality reduction algorithms (see Fig. S1 in the SI). Unfortunately, they both have a tuneable parameter that makes them less handy than PCA or ncMCE. It is worth mentioning that, when the value of this parameter generates a neighbourhood proximity graph with a tree-like structure from which the data is to be embedded (like the basis of ncMCE), these algorithms provide the best separation between the Japanese groups (see Fig. S2). This result suggests that the hidden manifold structure of this dataset has an intrinsic tree-like, hierarchical shape.

Colouring individuals by ethnicity in ncMCE's projection, as shown in Fig. 4c, revealed that the separation provided by this nonlinear approach was consistent with the phylogenetic differences between and the distant geographic locations of Japanese from Tokyo (the JPT and JP-ML ethnic groups) and Japanese Ryukyuan from Okinawa (the JP-RK ethnic group). We did not expect that <1% missing values would dramatically affect the result of PCA. Yet, this observation is especially relevant when merging data from different platforms or working with ancient DNA and should represent an important point to consider in future studies.

**Figure 5:**
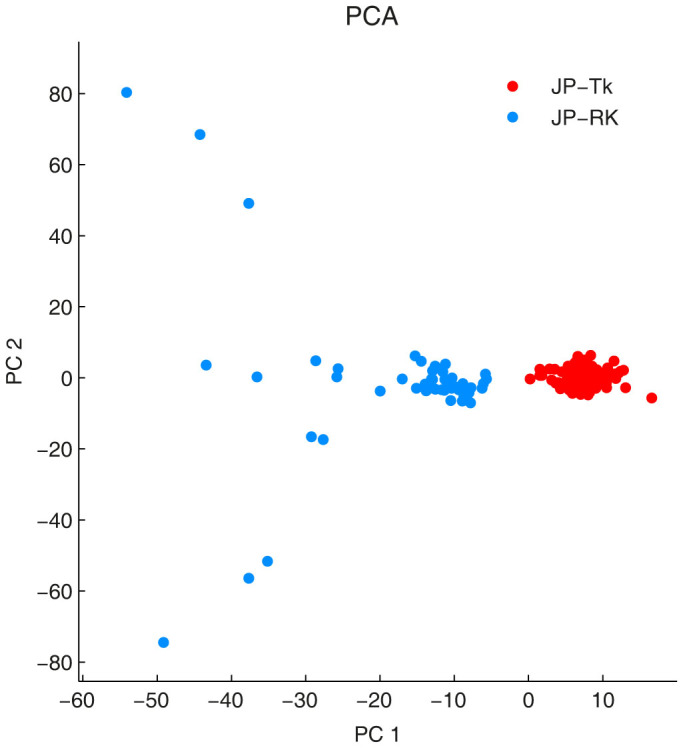
Linearisation of the Japanese dataset by substitution of the missing values. The missing values in the genotype matrix of Japanese individuals were substituted with the mode of each specific SNP to remove the nonlinear perturbations of this dataset and allow PCA to identify sub-groups, Tokyotas or JP-Tk, and Okinawans or JP-RK, that ncMCE was able to identify using the original data.

We wanted to ensure that the groups identified in the Japanese dataset by ncMCE were genetically meaningful and that the clustering was not based on missing data (i.e., a group of individuals with more missing SNP values and a group with fewer). For this analysis, we substituted the missing SNP values of an individual with the mode of that particular SNP across individuals as in the rest of this paper (the analysis was also repeated using other centrality measures like the mean or median, which are reasonable missing data imputation approaches when the proportion of missing values is very small in relation to the complete dataset as pointed out in the Methods) and applied PCA over the modified matrix (see Fig. 5 and Figs. S4a,b respectively). Surprisingly, as shown in Fig. 5 for the case of the mode, this substitution linearised the Japanese dataset, and PCA was able to detect the two groups identified by ncMCE using the original genotype matrix, indicating that the missing values in the Japanese dataset introduced a gap in the *continuity* of the multidimensional data structure, therefore causing a significant nonlinear perturbation in this dataset. This result confirmed that the clustering obtained was not an algorithmic artefact and that ncMCE could reveal patterns hidden by different sources of nonlinearity in the data, such as intricate phylogenetic relationships (see the case of the Malay population in Fig. 3b) or noise due to missing information (see the case of the Japanese individuals in Figs. 4 and 5). Additionally, this supports the complementarity of PCA and nonlinear techniques, wherein each technique mines different characteristics of the data being analysed.

**Figure 6:**
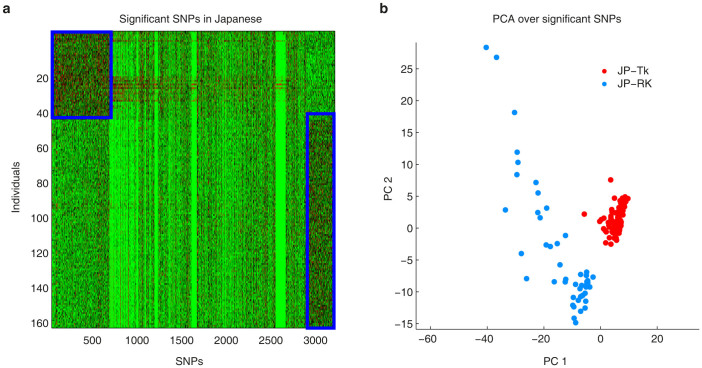
Mann–Whitney non-parametric statistical test confirmed ncMCE's sub-cluster detection. Extraction of the SNPs that most significantly differentiated between members of the sub-groups identified by ncMCE in the Japanese population (p ≤ 0.01) confirmed what ncMCE found: the presence of two sub-groups of individuals with clear genetic differences (a). The heat map shows the log_10_(1 + *SNP value*), in which the SNP values can be 0 (homozygous wild-type), 1 (heterozygous wild-type), 2 (homozygous variant type) or 3 (missing data). The SNPs are subdivided in a first set with high average values, in the top-left corner of the heat map, characterising the first cluster of individuals. The second set, in the bottom-right corner, has also high average values and characterises the other cluster. Note that the genetic variants in the first or the last set of SNPs make the two groups genetically different. Interestingly, the PCA projection of the Japanese individuals, which considered only the significant SNPs extracted from the original genotype matrix, revealed the two groups that ncMCE identified (b). PCA could not detect these groups upon application to the original dataset (Fig. 4a).

The results obtained from the Japanese dataset were further validated by performing a Mann–Whitney non-parametric statistical test over the original genotype matrix (no substitutions of missing value data with mode, mean or median) to detect the SNPs that were most significant for differentiating between members of the two groups identified by ncMCE and the other nonlinear dimensionality reduction techniques (p ≤ 0.01, see the Methods for more details). A heat map of individuals (vertical axis) and the detected, more significant SNPs only (horizontal axis), already suggests that the samples could be separated into two groups (Fig. 6a). Interestingly, the application of PCA to the significant SNPs alone permitted the detection of the two groups that ncMCE had identified in both situations (Fig. 6b) and that PCA had not been able to uncover using the original genotype matrix (Fig. 4a). These results also hold if Benjamini correction is applied to form a more stringent list of significant SNPs (see Figs. S5a and S5b respectively). This important result indicates that if the impact of the missing values on the data structure is minimised by elimination of non-discriminative features, the nonlinear structure hidden in the dataset is linearised, thereby rending this structure visible to PCA. In the SI, we show an example of what researchers can do once a set of interesting SNPs, like the above mentioned, have been identified. This kind of downstream analysis can aid in, for instance, the understanding of the origins of structure in population genetic datasets.

The above interesting results suggested that ncMCE was able to cope with poorly pre-processed data and detect differences between the Tokyota and Okinawan. PCA was also able to achieve this but only when the impact of the missing values is minimised by elimination of non-discriminative features or when the nonlinear structure hidden in the dataset is linearised by missing data imputation.

## Discussion

The amount of genetic data available to researchers today requires powerful techniques that facilitate the rapid interpretation of this valuable information. PCA's simplicity and computational speed make it one of the preferred statistical tools for analysing GWAS data. PCA's reduction of data to projections along axes of great variation lets us analyse the differences between individuals in case-control studies or population genetics data^5^. There are, however, some cases (e.g., the presence of nonlinearity in the data) in which PCA cannot reveal the presence of important patterns, and for these situations, we suggest that ncMCE be adopted as an auxiliary tool for PCA. Additionally, situations in which the data may be characterised by different linear and nonlinear patterns coexisting in the same multidimensional space, may benefit from the exploitation of the complementarity of PCA and ncMCE.

As shown in the examples above, ncMCE can identify structure within the datasets in which the differences between individuals are small. In addition, given that ncMCE relies on an MST, its orderings and the phylogenetic tree structure agree substantially, which is a very interesting and useful feature. ncMCE matches PCA in terms of computational speed and algorithmic simplicity. Both approaches can handle large numbers of individuals characterised by a massive number of features in a matter of minutes and the steps followed by these approaches for projecting data onto lower dimensions are simple, yet powerful.

The power to identify structured groups in genetic data for which PCA is unable to do so (even when more than two dimensions are considered, as we verified in all the datasets presented here), or in a complementary manner to PCA's results, renders the ncMCE approach as an important companion to PCA. ncMCE can guide researchers in their quest for intriguing sample relationships, which were invisible to linear approaches but were identified here, especially when the data is not correctly pre-processed or adjusted.

The detection of hidden nonlinear relations between individuals and representation of phylogenetic population relationships are points of strength and utility for ncMCE. To conclude, the results obtained here from the genetic data, together with the range of different fields within which ncMCE has been successfully applied^8^,^9^,^11^,^12^,^13^, suggest that this machine learning approach is, in general, a good option for detecting nonlinear multidimensional relationships in data mining and pattern recognition studies.

## Methods

### Data

The genotype data used in this paper corresponds to the Pan-Asian SNP Consortium Database (PanSNPdb)^16^. PanSNPdb comprises 75 populations (71 Pan-Asian and 4 from the HapMap Project) with 1928 individuals and 54,794 SNPs on autosomal chromosomes. The raw version of this dataset (available at http://www4a.biotec.or.th/PASNP/Download) was converted to the TPED format using a PERL script made available by the Harappa Ancestry Project at http://www.harappadna.org/2011/02/23andme-conversion-to-ped/. Finally, the corresponding BED, BIM and FAM files were generated using PLINK^22^ for further analysis in R.

### Genotype matrix

The PCA and ncMCE methods were applied to a genotype matrix, in which individuals were listed in rows and SNPs were listed in columns. The genotype matrix was generated using the R package SNPRelate (http://cran.r-project.org/package=SNPRelate). After loading the BED, BIM and FAM files that represented the PanSNPdb dataset, SNPRelate generated the genotype matrix using its function snpgdsGetGeno. This matrix was later exported to CSV format for further processing in MATLAB. For more details, we refer the reader to the documentation of the package.

### PCA and ncMCE

PCA and ncMCE MATLAB implementations were used to obtain the results presented throughout this paper. The MATLAB and R implementations of ncMCE are available at https://sites.google.com/site/carlovittoriocannistraci/.

### Clustering quality

To measure cluster quality over dimensions 1 and 2, we used the so called concordance score (C-score). C-score measures the ability of a clustering technique to separate individuals into their corresponding populations or ethnic groups over a single dimension. The C-score ranges from 0 to 1, where 0 corresponds to no population structure and 1 corresponds to a perfect ordering of individuals, in which populations or ethnic groups appear one after the other with no individuals belonging to one, mixed with the other. Formally, the C-score over dimension *d* is defined as^12^: 
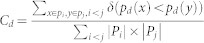
where *P_i_* is the set of individuals in population or ethnic group *i*, |*P_i_*| is the size of this population, *p_d_*(*x*) is a 1D projection of individual *x* over dimension *d* and *δ*(*cond*) is 0 or 1 depending on whether *cond* is *False* or *True*, respectively.

### Phylogenetic tree construction

Phylogenetic trees were constructed by averaging the SNPs of all individuals within a population or ethnic group in order to generate a representative sample. The representative samples were then hierarchically clustered according to an unweighted average distance to finally build the dendrograms shown in the figures throughout the article.

### Missing data imputation

Each individual in the above described genotype matrix was represented by a set of 54,794 SNPs that could take the genotype values of 0 (homozygous wild-type), 1 (heterozygous), 2 (homozygous variant-type) or 3. The latter value represents missing data. To deal with them, we used a strategy frequently adopted when the proportion of missing values is relatively small with respect to the data size^23^ (in our case only 0.30% of the total SNP values are missing values in the HapMap dataset, 0.86% in the Malay, 0.37% in the Singaporean and 0.73% in the Japanese dataset). We substituted the missing values in each of the 54,794 SNPs by the mode of the given values for each specific SNP. For the case of the Japanese dataset, missing values were also substituted by the mean and median of each specific SNP.

### Detection of most significant SNPs and heat map construction

Provided that the two groups identified by ncMCE were reliable, we performed a Mann–Whitney non-parametric statistical test to identify SNPs that most significantly differentiated between members of these groups. Thus, we treated each SNP in the Tokyota group as a column vector and compared it against the same SNP in the Okinawan group with the rank-sum test to obtain a p-value reporting whether the contribution of this SNP to the separation of the two groups is significant or not. Only SNPs with p-values ≤ 0.01 were selected and sorted according to their p-values to produce a data matrix in which each row represented an individual and the extracted, significant SNPs were listed across the columns. Multiple testing corrections were not performed, which preserved any noisy features and avoided the introduction of biases toward certain more discriminative features during the PCA analysis (nevertheless, multiple testing Benjamini correction confirmed our results as shown in Fig. S5 and Supplementary File 2). The rationale was to test whether also in the presence of less significant features, the two-cluster pattern was strong enough to be detectable by a linear transformation. The constructed heat map (Fig. 6a) corresponded to the log_10_(1 + *SNP value*) of the above-mentioned matrix, in which a SNP value could be 0 (homozygous wild-type), 1 (heterozygous wild-type), 2 (homozygous variant type) or 3 (missing data). 3173 significant SNPs were extracted from the Japanese population, which mapped to 1016 unique genes.

## Author Contributions

G.A.L. envisaged the study. G.A.L. and C.V.C. designed and carried out the experiments with inputs from the other authors. All the authors analysed the results. The article was mainly written by G.A.L. with corrections from C.V.C., A.E., A.M. and T.R. T.R. supervised the study.

## Supplementary Material

Supplementary Information Supplementary Information

Supplementary Information Supplementary File 1

Supplementary Information Supplementary File 2

## Figures and Tables

**Figure 1:**
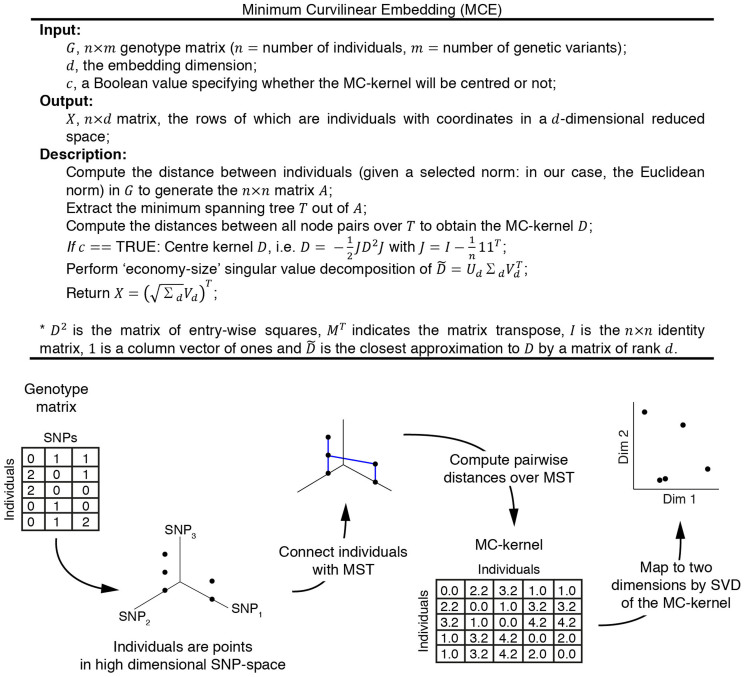
MCE computes distances between individuals (given a selected norm; in our case, the Euclidean norm) in *G* to generate the matrix of pairwise distances *A*. This matrix can be thought of as the adjacency matrix representation of a fully connected graph whose edges are weighted by inter-individual distances. A MST *T* is extracted from this graph, and distances between individuals are re-computed over it to obtain the MC-kernel *D*. In this paper, we used a version of MCE in which *D* is non-centred and the economy-size singular value decomposition is applied to it to determine the coordinates of each individual in a space of dimension *d*. This version of MCE is also known as ncMCE. The power of this approach relies on the MC-kernel. The MST *T* is a graph that extracts a greedy path that summarises the main relational information between the features of the dataset. This graph avoids noise and spurious information and emphasises the nonlinear relationship between the most representative and informative features of the data samples^8^,^9^,^15^,^24^.
