# Supplementary material for: Highlighting nonlinear patterns in population genetics datasets
Source: Sci Rep. 2015 Jan 30;5:8140. doi: 10.1038/srep08140 (PMC4311249; doi:10.1038/srep08140)
Supplement: Supplementary Information [file srep08140-s1.pdf]

SUPPLEMENTARY INFORMATION

Highlighting nonlinear patterns in population genetics datasets

Gregorio Alanis-Lobato[1,2,*,†], Carlo Vittorio Cannistraci[3,*,†], Anders Eriksson[1,4], Andrea Manica[4] & Timothy Ravasi[1,2,*]

[1]Integrative Systems Biology Laboratory, Biological and Environmental Sciences and Engineering Division, Computer, Electrical and Mathematical Sciences and Engineering Division, Computational Bioscience Research Center, King Abdullah University of Science and Technology (KAUST), Ibn Al Haytham Bldg. 2, Level 4, Thuwal 23955-6900, Kingdom of Saudi Arabia.

[2]Division of Medical Genetics, Department of Medicine, University of California, San Diego, 9500 Gilman Drive, La Jolla, CA 92093 USA.

[3] Biomedical Cybernetics Group, Biotechnology Center (BIOTEC), Technische Universität Dresden, Tatzberg 47/49, 01307 Dresden, Germany.

[4]Department of Zoology, University of Cambridge, Cambridge CB2 3EJ, England.

*To whom correspondence should be addressed. E-mail: gregorio.alanislobato@kaust.edu.sa (G.A.L.); kalokagathos.agon@gmail.com (C.V.C.); timothy.ravasi@kaust.edu.sa (T.R.)

[†]Joint first authors

Nonlinear dimensionality reduction techniques. The $n \times m$ genotype matrix G, with n individuals and m genetic variants or SNPs can be seen as a cloud of points (in this case individuals) lying near or on a low dimensional manifold embedded in a high dimensional feature space (in this case the space of SNPs). In order to represent the topological properties of such a manifold in low dimensions, several nonlinear dimensionality reduction techniques have been proposed, the majority of which construct a proximity graph by first connecting each point in the dataset with its k nearest neighbours and then projecting these points to a space of reduced dimensions by taking advantage of the structural properties of the graph.

In this paper, we used two different nonlinear dimensionality reduction approaches to compare their results with ncMCE's: Isomap[1] and Laplacian Eigenmaps[2]. The former constructs a distance matrix by measuring shortest-paths between points over the proximity graph and projects the points to low dimensions by multidimensional scaling of this distance matrix. The latter extracts the Laplacian from the proximity graph and recovers the low dimensional coordinates of the points by solving a generalised eigenvalue problem.

Note these two techniques have two free parameters: k, the number of nearest neighbours needed to construct the proximity graph and d, the dimension of embedding. We fixed the later to 2 but analysed the behaviour of different proximity graphs constructed with $k = 2\ to\ 31$ neighbours (see Fig. S1).

We quantified the success of the resulting two-dimensional projections by computing their C-score (see below for details and Fig. S2 for results).

Additional comments about ncMCE: As mentioned in the main article, ncMCE performs the embedding of the sample dissimilarities measured over their minimum spanning tree (MST). This novel MST-derived nonlinear measure, that we refer to as minimum curvilinearity (MC), gives rise to the MC-kernel. The MST is an acyclic graph with all the samples in a population as nodes, connected to each other by paths of minimum length. As a consequence, measuring distances over this graph emphasizes the separation between nodes far apart in the manifold and maintains or reduces the distances between nearby nodes, which produces a sort of gradual denoising and reveals nonlinear patterns hidden in the high-dimensional feature space[4].

The fact the in ncMCE the MC-kernel is not centred, poses the risk that this matrix is not always positive semi-definite and that its eigenvalues can be negative (with consequences in the projection to low dimensions). However, in practice, kernels that do not satisfy Mercer's condition (positive semi-definiteness of the kernel) can still be used as soon as they convey the intuitive idea of similarity[5], as in the case of Isomap's kernel[1,6] and some kernels used in kernel PCA, like the famous Sigmoid kernel[7].

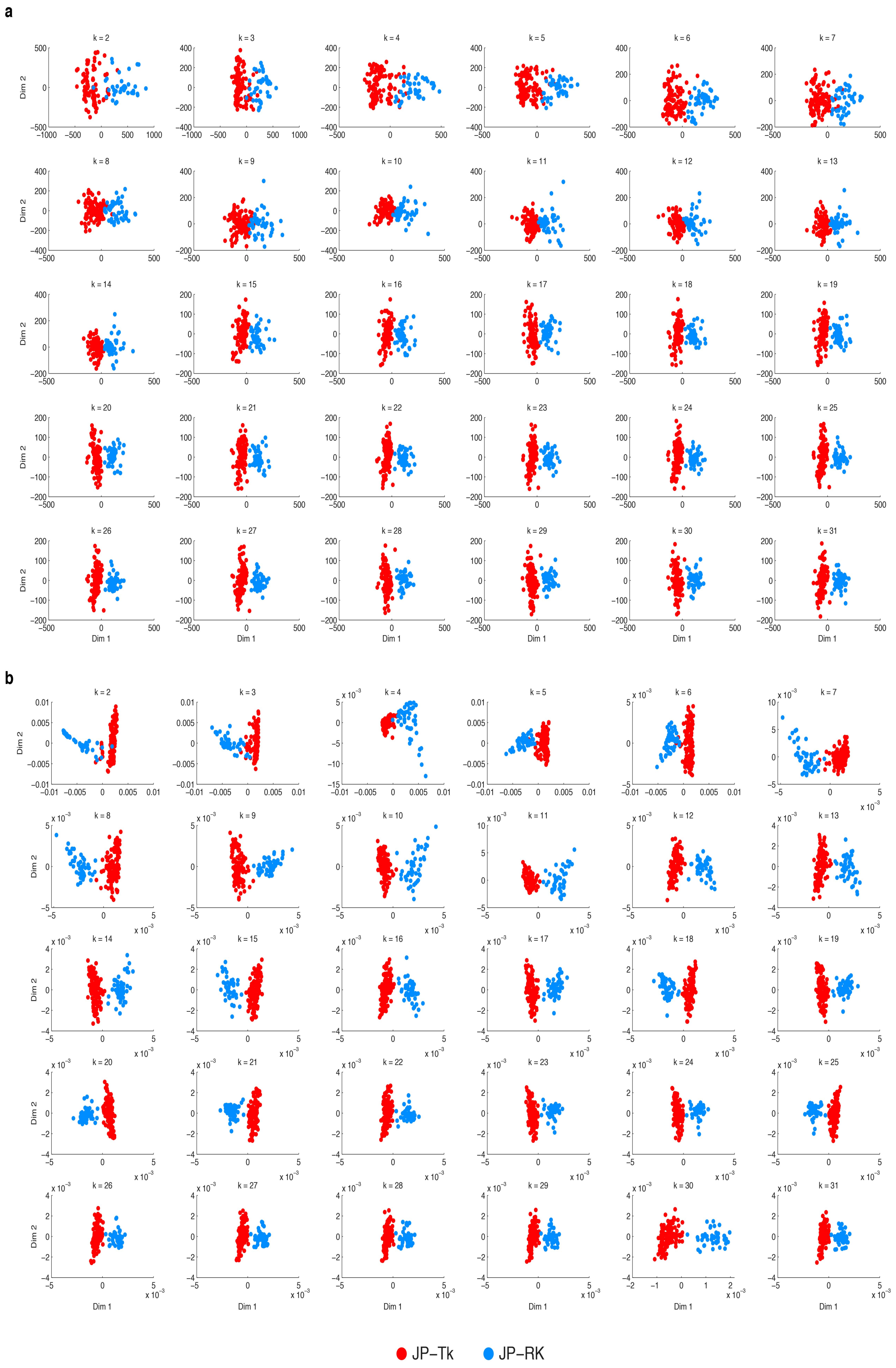

Figure S1. Nonlinear dimensionality reduction approaches applied to the Japanese population. (a) Isomap and **(b)** Laplacian Eigenmaps, nonlinear dimensionality reduction techniques, confirm the two subgroups found by ncMCE in the Japanese dataset: Tokyotas (JP-Tk) and Okinawans (JP-RK).

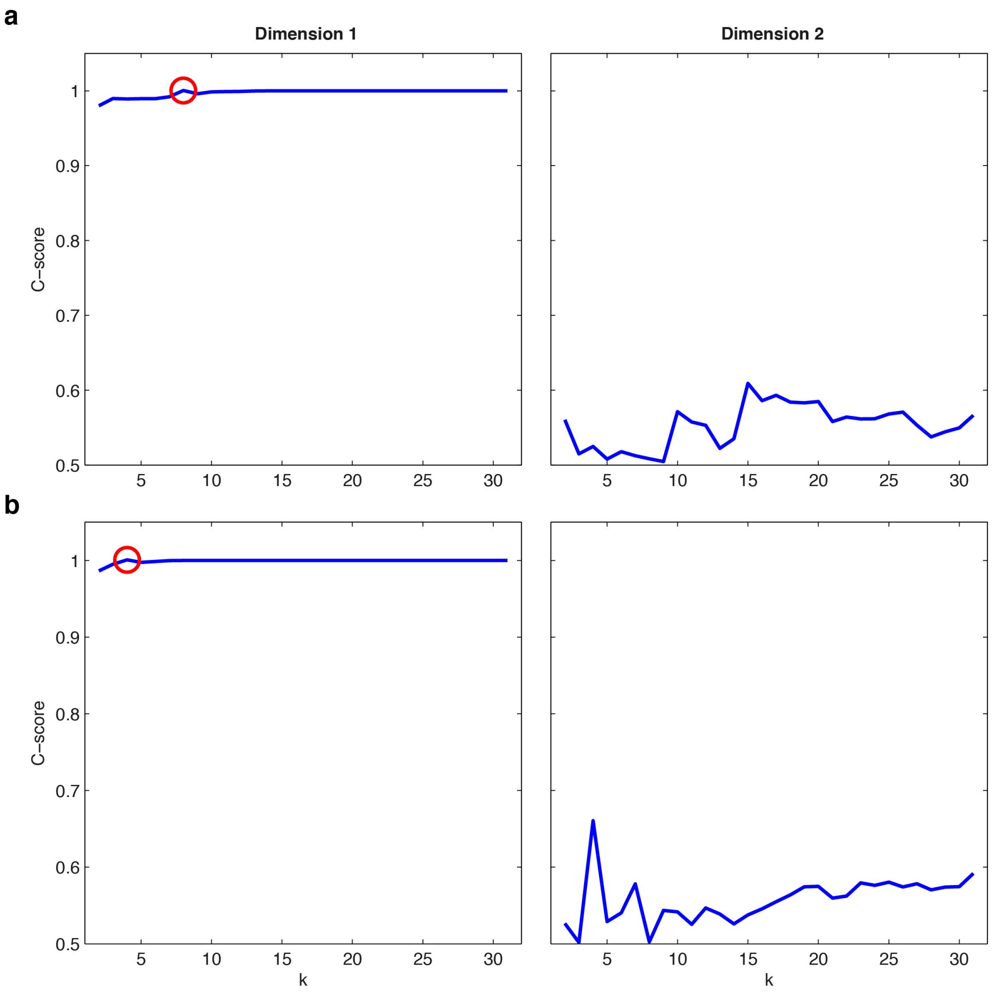

Figure S2. C-score indicating clustering quality over dimensions 1 (left) and 2 (right) in the Japanese population. (a) Isomap and **(b)** Laplacian Eigenmaps. Notice that the best C-score (red circle) is attained for a very low k, which means that a proximity graph with a tree-like structure, like ncMCE's basis, is preferred for providing a good discrimination between the two clusters.

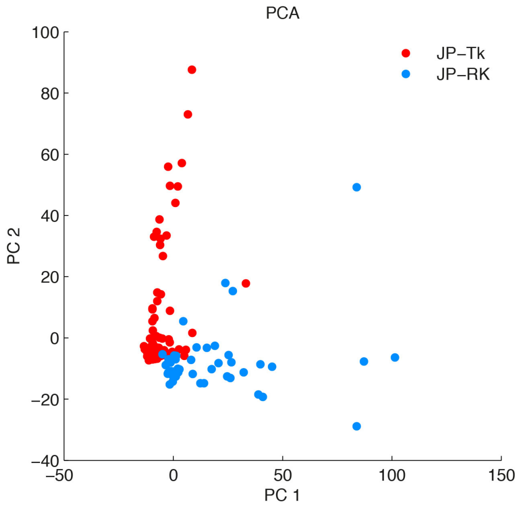

Figure S3. PCA applied to the Japanese population without substitution of missing values. PCA is unable to find the two clusters that ncMCE found on the original and unadjusted data matrix.

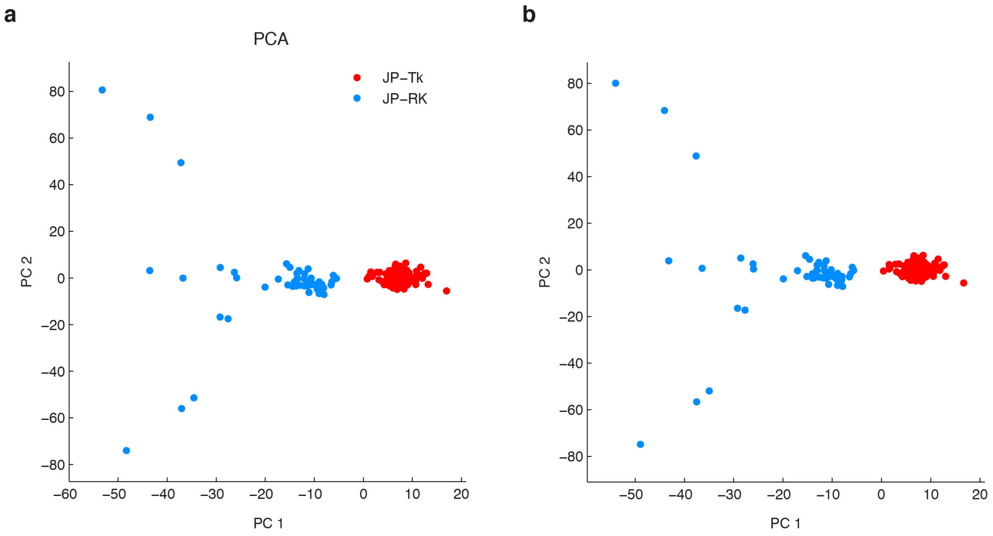

Figure S4. Linearisation of the Japanese dataset by substitution of missing values. Substituting missing values per SNP with mean **(a)** or median **(b)** linearises the Japanese dataset and allows PCA to find the two clusters that ncMCE found on the original and unadjusted data matrix (Fig. 4b,c in the main article).

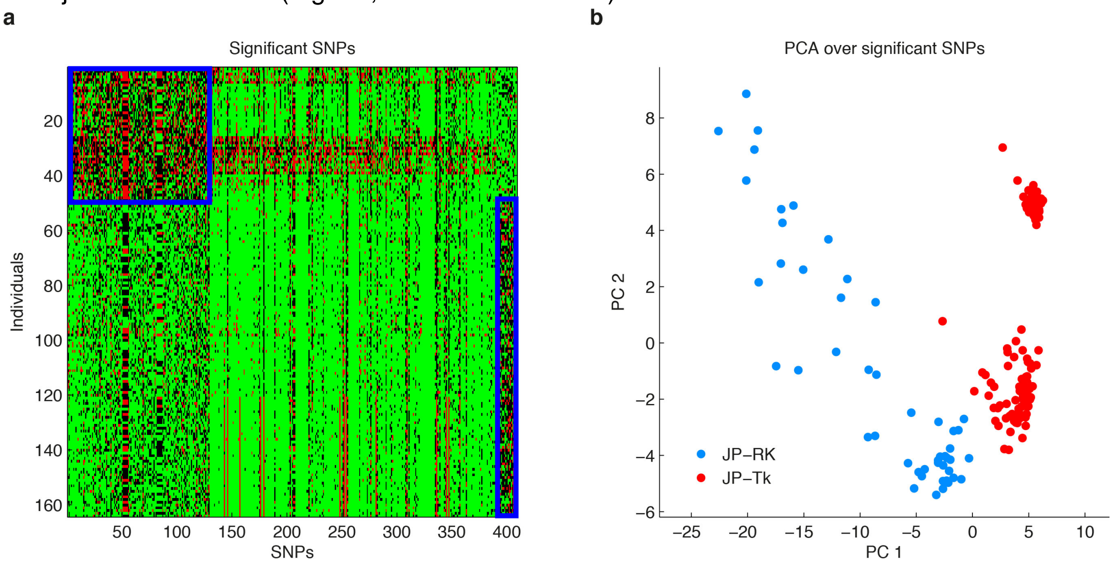

Figure S5. Mann–Whitney non-parametric statistical test with Benjamini correction confirmed ncMCE's sub-cluster detection. Extraction of the SNPs that most significantly differentiated between members of the sub-groups identified by ncMCE in the Japanese population ($p \leq 0.01$ after Benjamini correction) confirmed what ncMCE found: the presence of two sub-groups of individuals with clear genetic differences **(a)**. The heat map shows the $\log_{10}(1 + SNP\ value)$, in which the SNP values can be 0 (homozygous wild-type), 1 (heterozygous wild-type), 2 (homozygous variant type) or 3 (missing data). The SNPs are subdivided in a first set with high average values, in the top-left corner of the heat map, characterising the first cluster of individuals. The second set, in the bottom-right corner, has also high average values and characterises the other cluster. Note that the genetic variants in the first or the second set of SNPs make the two groups genetically different. Interestingly, the PCA projection of the Japanese individuals, which considered only the significant SNPs extracted from the original genotype matrix, revealed the two groups that ncMCE identified **(b).** PCA could not detect these groups upon application to the original dataset (Fig. 4a in the main article).

Example downstream analysis of the SNPs that most significantly differentiate between Japanese ethnic groups. We explored the role of the genes to which the significant SNPs (responsible for the separations amongst the Japanese populations) mapped (see description of the SNP-to-gene mapping below). After performing a functional enrichment analysis of the respective gene list (see the Supplementary Files 1 and the SNP-to-gene mapping details below), we found that the genes are significantly involved in pathways associated with diseases, such as Alzheimer's (p = 1.23E–8, Benjamini; p = 1.59E-7, Bonferroni; see the Methods for details on the meaning of these p-values), certain cardiomyopathies (p < 2.228E-9, Benjamini; p < 2.74E-8, Bonferroni) and certain types of cancer (p < 2.3E-4, Benjamini; p < 0.008, Bonferroni) or with neuronal activity (p < 2,26E–10, Benjamini; p < 2.26E-9, Bonferroni) and melanogenesis (p = 3.74E–6, Benjamini; 8.59E-5; Bonferroni). The genes are also significantly involved in the bioprocesses that govern neurogenesis (p < 3.45E-4, Benjamini; p < 0.006, Bonferroni) and cell proliferation (p = 3.06E–4, Benjamini; p = 0.005, Bonferroni). If the p-values used to select the most significant SNPs are Benjamini-corrected, the results are the same although the number of genes is reduced (see Supplementary File 2 and the SNP-to-gene mapping details below).

The fact that these diseases and pathways tend to be more present in elders and are related to aging processes, readily drew our attention to the Okinawa Centenarian Study[8,9], a research project based on reliable age verification data, with the goal of understanding why Okinawans present such as an exceptional longevity and represent the ethnic group with the world's highest ratio of centenarians (40–50 per 100,000 persons[8]). To our surprise, one of the findings of this study was that Okinawan elders experience a slower age decline and a delay or complete avoidance of the diseases associated with aging, such as Alzheimer's, cardiovascular disorders and cancer compared to other Japanese ethnic groups[8,10]. In addition, Okinawan centenarians possess HLA alleles that lower their risk of developing inflammatory and autoimmune disorders[10]. Two genes from the HLA family are part of the list of significant genes identified in this study (see the genes highlighted in yellow in the Supplementary File S1).

Moreover, a recent study[11] may explain why we found pathways and processes associated with neuronal activity, neurogenesis, melanogenesis and cell proliferation in general. Katsimpardi and colleagues found that restoring the functionality of age-related processes like blood flow and neural stem cell production counteracted the negative effects of aging in mice[11]. They also discovered that administration to old mice of a member of the TGF-β protein family, a family of factors that decreases with aging, reversed the age-related decline of neurogenesis and contributed to vascular remodelling[11]. Two members of this family of proteins are part of the list of significant genes identified in this study (see the genes highlighted in green in the Supplementary File S1).

SNP to gene mapping. The SNP-to-gene mapping was performed using Barts Cancer Institute's SNPnexus web database[12] with default parameters. We batch-queried the GRCh37/hg19 assembly with the dbSNP identifiers of all SNPs in the PanSNPdb dataset and with the dbSNP identifiers of the significant SNPs found in the Japanese population.

Gene functional analysis. DAVID Bioinformatics Resources 6.7[13] was used to find that the genes that separated the Japanese population into two groups were significantly associated with age-related diseases and pathways (1016 genes listed in Supplementary File 1). It is important to mention that, for this analysis, our background was set to be the collection of all genes to which the whole set of SNPs in the PanSNPdb dataset map: 54,794 SNPs that mapped to 6320 genes. This was done in order to produce more conservative p-values. In fact, a general guideline for enrichment analyses is to use a narrowed-down list of genes instead of all genes in the human genome as a background[4,13,14]. The reported p-values are the result of a modified, more conservative Fisher exact test in which it is checked whether the user gene list (the set of 1016 genes) is specifically enriched in age-related diseases and pathways than random chance compared to the background (the set of 6320 genes).

Supplementary File 1. Excel file with the significant genes involved in the separation of the Japanese population into two groups (no multiple testing correction was applied to detect the SNPs that map to these genes) along with pathways and bioprocesses that are enriched for these genes.

Supplementary File 2. Excel file with the significant genes involved in the separation of the Japanese population into two groups (Benjamini correction was applied to detect the SNPs that map to these genes) along with pathways and bioprocesses that are enriched for these genes.

REFERENCES

1. Tenenbaum, J. B., de Silva, V. & Langford, J. C. A global geometric framework for nonlinear dimensionality reduction. *Science (80-.).* **290,** 2319–2323 (2000).
2. Belkin, M. & Niyogi, P. Laplacian eigenmaps and spectral techniques for embedding and clustering. *Adv. Neural Inf. Process. Syst.* **14,** 585–591 (2001).
3. Zagar, L. *et al.* Stage prediction of embryonic stem cell differentiation from genome-wide expression data. *Bioinformatics* **27,** 2546–2553 (2011).
4. Cannistraci, C. V., Alanis-Lobato, G. & Ravasi, T. Minimum curvilinearity to enhance topological prediction of protein interactions by network embedding. *Bioinformatics* **29,** i199–i209 (2013).
5. Zhang, L., Zhou, W. & Jiao, L. Hidden space support vector machines. *IEEE Trans. Neural Networks* **15,** 1424–1434 (2004).
6. Lee, J. A., Lendasse, A. & Verleysen, M. Nonlinear projection with curvilinear distances: Isomap versus curvilinear distance analysis. *Neurocomputing* **57,** 49–76 (2004).
7. Lin, H.-T. & Lin, C.-J. *A Study on Sigmoid Kernels for SVM and the Training of non-PSD Kernels by SMO-type Methods.* 1–32 (2003).
8. Willcox, D. C., Willcox, B. J., Hsueh, W.-C. & Suzuki, M. Genetic determinants of exceptional human longevity: insights from the Okinawa Centenarian Study. *Age (Omaha).* **28,** 313–332 (2006).
9. Willcox, D. C., Willcox, B. J., Todoriki, H. & Suzuki, M. The Okinawan diet: health implications of a low-calorie, nutrient-dense, antioxidant-rich dietary pattern low in glycemic load. *J. Am. Coll. Nutr.* **28,** 500S–516S (2009).
10. Takata, H., Suzuki, M., Ishii, T., Sekiguchi, S. & Iri, H. Influence of major histocompatibility complex region genes on human longevity among Okinawan-Japanese centenarians and nonagerians. *Lancet* **2,** 824–826 (1987).
11. Katsimpardi, L. *et al.* Vascular and Neurogenic Rejuvenation of the Aging Mouse Brain by Young Systemic Factors. *Science (80-.).* **344,** 630–634 (2014).
12. Chelala, C., Khan, A. & Lemoine, N. R. SNPnexus: a web database for functional annotation of newly discovered and public domain single nucleotide polymorphisms. *Bioinformatics* **25,** 655–61 (2009).
13. Huang, D. W., Sherman, B. T. & Lempicki, R. Systematic and integrative analysis of large gene lists using DAVID bioinformatics resources. *Nat. Protoc.* **4,** 44–57 (2009).
14. Huang, D. W., Sherman, B. T. & Lempicki, R. A. Bioinformatics enrichment tools: paths toward the comprehensive functional analysis of large gene lists. *Nucleic Acids Res.* **37,** 1–13 (2009).
